# Correction: Tissue-specific shaping of the TCR repertoire and antigen specificity of iNKT cells

**DOI:** 10.7554/eLife.56997

**Published:** 2020-03-18

**Authors:** Rebeca Jimeno, Marta Lebrusant-Fernandez, Christian Margreitter, Beth Lucas, Natacha Veerapen, Gurdyal S Besra, Franca Fraternali, Jo Spencer, Graham Anderson, Patricia Barral

Jimeno R, Lebrusant-Fernandez M, Margreitter C, Lucas B, Veerapen N, Besra GS, Fraternali F, Spencer J, Anderson G, Barral P. 2019. Tissue-specific shaping of the TCR repertoire and antigen specificity of iNKT cells. *eLife* **8**:e51663. doi: 10.7554/eLife.51663.

Published 16, December 2019

Gavin Kelly was originally omitted from the authors list. However, he contributed to RNAseq analyses and we believe he should be included as an author. All authors have agreed to this change.

**New authors list**: Rebeca Jimeno, Marta Lebrusant-Fernandez, Christian Margreitter, Beth Lucas, Natacha Veerapen, **Gavin Kelly,** Gurdyal S. Besra, Franca Fraternali, Jo Spencer, Graham Anderson & Patricia Barral

**Original authors list**: Rebeca Jimeno, Marta Lebrusant-Fernandez, Christian Margreitter, Beth Lucas, Natacha Veerapen, Gurdyal S. Besra, Franca Fraternali, Jo Spencer, Graham Anderson & Patricia Barral

**Details for the omitted author:**

**Gavin Kelly**

Bioinformatics and Biostatistics Science Technology Platform, The Francis Crick Institute, London, UK.

**Contribution**: Data curation, Formal analysis

**Competing interests**: No competing interests declared

**Revised Acknowledgements:**

This work was funded by the Medical Research Council (grant to PB MR/L008157/1); RJ was supported by a Marie Curie Intra-European Fellowship (H2020-MSCA-IF-2015–703639); ML-F is funded by a Francis Crick Institute-King’s College London studentship; GA and BL are supported by a Medical Research Council Programme Grant (DKAA.RRAK18742). We thank the Science Technology Platforms from the Francis Crick Institute (which receives its core funding from Cancer Research UK, the UK Medical Research Council, and the Wellcome Trust). We acknowledge the NIH Tetramer Core Facility for provision of CD1d tetramers.

**Original Acknowledgements:**

This work was funded by the Medical Research Council (grant to PB MR/L008157/1); RJ was supported by a Marie Curie Intra-European Fellowship (H2020-MSCA-IF-2015–703639); ML-F is funded by a Francis Crick Institute-King’s College London studentship; GA and BL are supported by a Medical Research Council Programme Grant (DKAA.RRAK18742). We acknowledge the NIH Tetramer Core Facility for provision of CD1d tetramers.

The article has been corrected accordingly.

